# Investigation of the Influence of Anti-Solvent Precipitation Parameters on the Physical Stability of Amorphous Solids

**DOI:** 10.3390/molecules29061275

**Published:** 2024-03-13

**Authors:** Zunhua Li, Zicheng Gong, Bowen Zhang, Asad Nawaz

**Affiliations:** 1 College of Chemistry and Bioengineering, Hunan University of Science and Engineering, Yongzhou 425199, China; 19574455725@163.com (Z.G.); 007298@yzu.edu.cn (A.N.); 2 School of Chemical Engineering and Technology, Tianjin University, Tianjin 300072, China; bowen_667@tju.edu.cn

**Keywords:** amorphous solid, physical stability, pair distribution function, reduced crystallization temperature, principal component analysis

## Abstract

Amorphous solids exhibit enhanced solubility and dissolution rates relative to their crystalline counterparts. However, attaining optimal bioavailability presents a challenge, primarily due to the need to maintain the physical stability of amorphous solids. Moreover, the precise manner in which precipitation parameters, including the feeding rate of the anti-solvent, agitation speed, and aging time, influence the physical stability of amorphous solids remains incompletely understood. Consequently, this study aimed to investigate these three parameters during the precipitation process of the anticancer drug, nilotinib free base. The physical stability of the resultant samples was evaluated by employing characterization techniques including powder X-ray diffraction (PXRD), differential scanning calorimetry (DSC), focused beam reflectance measurement (FBRM), and data analysis methods such as pair distribution function (PDF), reduced crystallization temperature (R_c_), and principal component analysis (PCA). This study’s findings indicated that amorphous solids exhibited the greatest physical stability under particular conditions, namely a feeding rate of 5 mL/min, an agitation speed of 500 rpm, and an aging time of 10 min. Furthermore, the physical stability of the amorphous solids was primarily influenced by particle size and distribution, molecular interactions, microstructure, surface area, and interfacial energy. Notably, the parameters involved in the anti-solvent precipitation process, including the feeding rate of the anti-solvent, agitation speed, and aging time, exerted a significant impact on these factors. Consequently, they directly affected the physical stability of amorphous solids. Hence, this study comprehensively elucidated the mechanistic influence of these operational parameters on the physical stability of amorphous solids during the anti-solvent precipitation process.

## 1. Introduction

Recently, there has been significant attention given to amorphous solids due to their enhanced solubility and dissolution rates in comparison with their low aqueous solubility crystalline form [1,2]. However, amorphous solids are unstable and can transform into a crystalline form, reducing the solubility and bioavailability of active pharmaceutical ingredients (APIs) [3,4]. The development of amorphous solids poses challenges due to their inadequate physical stability. Polymeric amorphous solid dispersions, co-amorphous systems, and polymeric amorphous salts are among the extensively investigated formulation strategies that utilize the amorphous state of a drug to improve its solubility [5,6]. While these methods can indirectly enhance the stability of amorphous solids, it is equally important to develop amorphous solids that are inherently stable. Therefore, it is crucial to utilize appropriate preparation techniques to attain physically stable amorphous solids and to establish suitable assessment methods for evaluating their physical stability. Moreover, the mechanism by which precipitation parameters affect amorphous physical stability has not been thoroughly studied.

Hence, in terms of the production of physically stable amorphous solids, various methods for amorphous solids preparation, including grinding, spray drying, anti-solvent precipitation, and hot melt extrusion, may influence the microstructure and stability of amorphous solids [7]. Anti-solvent precipitation was the most commonly used method for preparing amorphous solids, and it was favored due to four main reasons. Firstly, it effectively governed the crystallization process, thus averting the formation of crystalline products. Secondly, the precise manipulation of parameters such as the feeding rate of the anti-solvent and agitation speed allowed for precise control over particle morphology and size. Thirdly, when compared with alternative amorphous solids preparation techniques, the anti-solvent precipitation method boasted simplicity in operation, minimal equipment requirements, and ease of scaling up for industrial production. Lastly, the anti-solvent precipitation method typically incurs lower costs compared with alternative methods, thus enhancing its competitiveness in industrial settings.

Moreover, the physical stability of amorphous solids was affected by a variety of factors, including particle size and distribution, molecular interactions, microstructure, surface area, and interface energy, and the anti-solvent precipitation parameters can influence these factors. For example, the amorphous solid preparation parameters employed in anti-solvent precipitation methods, including residual solvent, drying time, and anti-solvent/solvent ratio, also have an impact on the physical stability of the obtained amorphous solids [8]. In addition, the type of anti-solvent also affects the preparation of the amorphous solids. For instance, the use of deionized water as the anti-solvent resulted in the formation of amorphous telmisartan particles, whereas the use of a mixture of deionized water and dimethyl sulfoxide (DMSO) as the anti-solvent led to the production of the crystalline form [9]. In addition, the feeding rate of the anti-solvent also has an impact on the preparation of amorphous solids; for example, using a higher feeding rate of the anti-solvent results in increased supersaturation, therefore obtaining the amorphous form of disodium guanosine 5′-monophosphate, while the formation of hydrate crystals occurred at a slower feeding rate of the anti-solvent [10]. This is because the feeding rate of the anti-solvent played a crucial role in regulating the precipitation behavior of the amorphous solids, thereby promoting the formation or physical stability of the amorphous solids. Moreover, the feeding rate of the anti-solvent significantly impacted the interaction between the anti-solvent and the solvent, influencing both the nucleation and growth rates of the amorphous solids, ultimately determining their physical stability. Additionally, agitation speed was another vital factor in the amorphous solid preparation. For instance, at slower agitation speeds, candesartan cilexetil undergoes self-induced nucleation, leading to the formation of crystalline structures. However, at higher agitation speeds, the prenucleation clusters transition into an amorphous form [11]. Furthermore, the aging time was also a crucial consideration for ensuring the physical stability of the amorphous solids, as these materials typically exist in a high-energy state with relatively low physical stability. Therefore, selecting an appropriate aging time was essential for achieving physically stable amorphous solids [12].

Furthermore, in regards to the aspect of evaluating the physical stability of the amorphous solids, it was demonstrated that combining the use of the pair distribution function (PDF, G(r)) and principal component analysis (PCA) has emerged as a valuable tool in recent years for accessing the disorder and physical stability of amorphous solids. For example, Li et al. showed that by combining the PDF and PCA, the amorphous solids of nilotinib free base exhibited the greatest degree of disorder and physical stability under specific precipitation conditions, including a 50 mL washing water volume, 18 h of drying time, and a 40 anti-solvent/solvent ratio [8]. Furthermore, a study was undertaken to examine the capacity of PDF and PCA in determining the optimal cryo-milling duration necessary to achieve the highest degree of disorder and stability in γ-indomethacin samples [13]. In addition, the reduced physical stability of amorphous piroxicam was ascribed to the heightened residual order identified in the amorphous solid samples, as also determined by the PDF and PCA [14]. These studies have shown that a lower G(r) value in an amorphous solid sample compared with other amorphous solid samples indicated a higher degree of disorder and a decreased likelihood of transforming into a crystalline form. This observation implies that amorphous solids with a higher degree of disorder exhibit higher physical stability [8,13,14,15,16]. Moreover, the reduced crystallization temperature (R_c_) values can also be utilized as a method for assessing the physical stability of the amorphous solids [17,18]. The R_c_ value serves as a normalized indicator of the extent to which a sample must be heated above its glass transition temperature (T_g_) for crystallization to occur spontaneously. The R_c_ value was utilized to evaluate the tendency of amorphous solids to undergo crystallization. The physical stability of an amorphous solid exhibits a positive correlation with its R_c_ value. A higher R_c_ value indicates a higher resistance to crystallization and provides increased protection against the formation of a crystalline form from an amorphous solid. An amorphous solid with a lower R_c_ value will undergo a more rapid transition into the crystalline form and exhibit reduced physical stability [8].

The crystalline Form A of nilotinib free base demonstrated low aqueous solubility (2 μg/mL in phosphate-buffered saline, pH 6.8) [19] and limited bioavailability (30% or less) [20]. Therefore, nilotinib free base has been classified as a biopharmaceutics classification system (BCS) class IV compound [21]. Consequently, several approaches have been devised to obtain the amorphous solid of nilotinib free base. These methods include the formation of an amorphous solid dispersion by spray drying with polymeric excipients [22], the fabrication of amorphous nanosuspension by an acid-base neutralization approach [23], the encapsulation into yeast glucan particles to prepare the amorphous solids [24], and the formulation of supersaturating formulations [20]. However, there is a scarcity of research on methods to enhance the physical stability of amorphous solids of nilotinib free base. For instance, our laboratory conducted a study on optimizing precipitation conditions to produce physically stable amorphous solids of nilotinib free base. This was achieved by using PDF analysis and the R_c_ value [8]. However, the aforementioned research only examined three preparation parameters (residual solvent, drying time, and anti-solvent/solvent ratio) considering the impact on the physical stability of nilotinib free base amorphous solids, which is insufficient.

Hence, the current study aimed to enhance the physical stability of amorphous solids of nilotinib free base by manipulating the other three precipitation conditions, including the feeding rate of the anti-solvent, the agitation speed, and the aging time. Furthermore, PDF analysis and R_c_ values continue to be used to evaluate the physical stability of amorphous solids. Furthermore, this study also examined the impact of precipitation conditions on the filtration rate in the precipitation process.

## 2. Results and Discussion

### 2.1. Effect of Feeding Rate

During the investigation of the feeding rate of the anti-solvent, the agitation speed was maintained at a constant rate of 500 rpm, while the aging time was fixed at 10 min, allowing only the feeding rate of the anti-solvent to vary.

#### 2.1.1. PDF Analysis and PCA of PXRD Data

**Figure 1:**
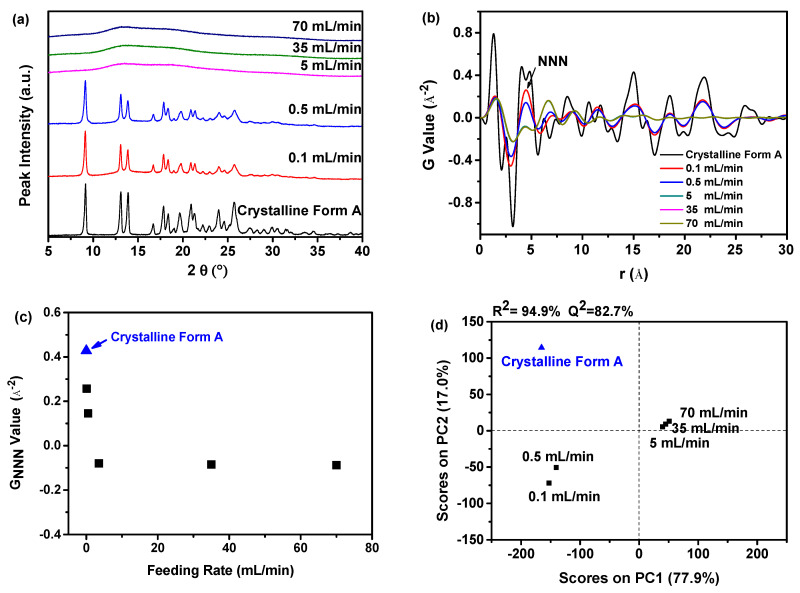
The influence of the feeding rate of the anti-solvent on the formation of nilotinib free base solid samples: (**a**) PXRD patterns; (**b**) PDF trace (the NNN represents the next nearest neighbor atoms in the nilotinib free base solid samples); (**c**) G_NNN_ value; (**d**) PCA scores plot (The R^2^ represents the goodness of fit, while the Q^2^ represents the goodness of prediction). The crystalline Form A was used as a reference sample; the agitation speed was fixed at 500 rpm, and the aging time was fixed at 10 min.

As illustrated in Figure 1a, the PXRD patterns of the dried samples obtained at feeding rates of 0.1 and 0.5 mL/min exhibited distinct peaks at 9.07°, 13.08°, 13.82°, 16.68°, 17.85°, 18.26°, 20.84°, 21.39°, 24.11°, and 25.22°, which corresponded to the crystalline Form A of nilotinib free base. These findings align precisely with the characteristic peaks of nilotinib free base Form A documented in the previous literature [25,26]. Nevertheless, when the feeding rates were set at 5, 35, and 70 mL/min, the dried samples solely exhibited the existence of a halo peak that corresponds to the amorphous state of a nilotinib free base. The distinction between crystalline and amorphous forms can be readily discerned through PXRD patterns. Nevertheless, distinguishing between various samples of amorphous solids based on PXRD patterns presents a challenge [27].

Hence, PDF analysis was utilized to convert the PXRD data and differentiate the differences among different amorphous solids, as illustrated in Figure 1b. During the analysis of the PDF trace for nilotinib free base, it was observed that the peak at the atomic distance (r = 4.62 Å) corresponds to the next nearest neighbor (NNN) peak, and this atomic distance represents the crystal lattice height of nilotinib free base in its crystalline Form A [28]. It is noteworthy that similar analyses have been conducted with other drugs, such as indomethacin, where it was similarly established that the NNN peak aligns with the molecular coordination sphere [29]. Consequently, the solid state of nilotinib free base, precipitated at feeding rates of 0.1 and 0.5 mL/min, demonstrated increased variations in the PDF trace and showed a higher peak height for the NNN peak in comparison with the amorphous solids produced at faster feeding rates of 5, 35, and 70 mL/min. The faster feeding rates resulted in the formation of more disordered amorphous solids, attributed to the generation of higher supersaturation. The elevated supersaturation led to the premature solidification of the resulting solids, preventing them from aligning correctly and impeding the formation of a crystalline structure, which entails a more orderly arrangement of molecules [30,31]. That is, the practical formation of an amorphous- or crystalline-form solid depends on the rate of crystallization at supersaturation in the anti-solvent [32,33]. Meanwhile, an analysis of the peak height of NNN revealed a minor variation in the amorphous solid samples when the feeding rate was adjusted from 5 to 70 mL/min, which means these three amorphous solid samples present slight variations in their degree of disorder.

The degree of disorder in these samples can be accurately evaluated through the G_NNN_ value, as depicted in Figure 1c. The G_NNN_ value of the samples precipitated at feeding rates of 0.1 and 0.5 mL/min was found to be higher in comparison with the samples precipitated at feeding rates of 5, 35, and 70 mL/min. Specifically, the G_NNN_ value reached its maximum when the feeding rate was 0.1 mL/min. Furthermore, there was a minor discrepancy in the G_NNN_ value across the three samples that were precipitated at a feeding rate exceeding 5 mL/min. The findings revealed that the samples produced at a slower feeding rate demonstrated a reduced degree of disorder in comparison with those produced at a faster feeding rate. Additionally, the three samples prepared at a feeding rate exceeding 5 mL/min exhibited minor fluctuations in the degree of disorder.

Furthermore, to effectively evaluate the difference between these samples, a PCA was performed using the previously mentioned PDF data, as shown in Figure 1d. The study findings indicated a high goodness of fit (R^2^) was achieved at 94.9%, suggesting a strong correspondence between the observed and predicted values. Furthermore, the predictive accuracy (Q^2^) was calculated to be 82.7%, indicating a high level of precision in forecasting future events. The results of the PCA analysis indicated that the fitting and prediction were of high quality. Upon comparing the samples obtained under various feeding rates with the reference sample (crystalline Form A), it was noted that they could be classified into three distinct groups. The initial group comprised the reference sample (crystalline Form A), while the second group encompassed the samples prepared at feeding rates of 0.1 and 0.5 mL/min. The third group consisted of the three samples prepared at feeding rates exceeding 5 mL/min. It is noteworthy that the samples prepared at the feeding rates of 0.1 and 0.5 mL/min exhibited distinct separation from the three samples prepared at feeding rates exceeding 5 mL/min in the PCA plot. The sample prepared at a 0.1 mL/min feeding rate exhibited the closest proximity to the reference sample (crystalline Form A) on the PC1. This suggests that, among all the samples prepared at different feeding rates, the sample prepared at a feeding rate of 0.1 mL/min showed the highest similarity to the reference sample (crystalline Form A) regarding the degree of disorder of the nilotinib free base molecule. This interpretation was consistent with previous studies [14,27].

Furthermore, previous research demonstrated that the impact of the feeding rate of the anti-solvent on the physical stability of amorphous solids primarily depended on the following mechanism: the feeding rate of the anti-solvent governed the extent of mixing between the anti-solvent and the solvent. An excessively rapid feeding rate can result in uneven mixing, leading to a local concentration gradient [34]. This, in turn, can impact the uniformity and stability of amorphous solids. Therefore, the experimental findings suggested that elevating the feeding rate to 5 mL/min resulted in the formation of a more physically stable amorphous solid in the present investigation.

#### 2.1.2. R_c_ Analysis of DSC Data

**Figure 2:**
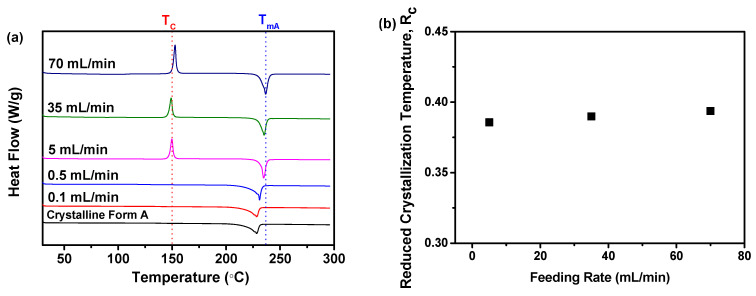
The influence of the feeding rate of the anti-solvent on the formation of nilotinib free base solid samples: (**a**) DSC curves (the T_C_ represents the crystallization temperature of the amorphous solid samples, while the T_mA_ represents the melting temperature of nilotinib free base crystalline Form A); (**b**) Reduced crystallization temperature, R_c_ value. (the crystalline Form A was used as a reference sample; the agitation speed was fixed at 500 rpm, and the aging time was fixed at 10 min).

The DSC analysis was performed to further validate the previously mentioned findings obtained from PXRD analysis. As illustrated in Figure 2a, the samples produced at varying feeding rates displayed a solitary endothermic peak, corresponding to the melting temperatures (T_m_) of nilotinib free base crystalline Form A (T_mA_, 235 °C) [25,26]. In addition, the T_mA_ of the samples precipitated at feeding rates of 0.1 and 0.5 mL/min was observed to be slightly lower than the T_mA_ of the samples precipitated at feeding rates exceeding 5 mL/min. Moreover, there was no notable variation in the T_mA_ value among the three samples prepared at a feeding rate exceeding 5 mL/min, despite the differences in the feeding rates employed.

Meanwhile, it was noted that the three samples prepared at a feeding rate exceeding 5 mL/min displayed an exothermic peak at 150 °C, which was ascribed to the transformation from the amorphous form to the crystalline form of nilotinib free base, which was consistent with the crystallization temperature (T_c_) of the nilotinib free base crystalline form A obtained in our previous study [8]. Furthermore, before performing the DSC measurement, it was confirmed by PXRD that the three samples precipitated at feeding rates exceeding 5 mL/min were amorphous solids. With the temperature rise during the DSC measurement, the amorphous solids transformed to crystalline Form A. However, the samples prepared at the feeding rates of 0.1 and 0.5 mL/min did not exhibit an exothermic peak, suggesting that these two samples were in a pure crystalline Form A before heating. There were no indications of amorphous solids in the samples prepared at the feeding rates of 0.1 and 0.5 mL/min, and therefore, no phase transformation from amorphous solid to crystalline Form A occurred during the heating process in the DSC measurement.

Furthermore, Figure 2b illustrated the reduced crystallization temperature (R_c_) value, which was found to be dependent on the feeding rate. The experiment results indicated that the samples prepared at feeding rates of 0.1 and 0.5 mL/min were unable to calculate the R_c_ value because the T_c_ value was absent in these two samples. In addition, the samples prepared at a feeding rate exceeding 5 mL/min showed only minor fluctuations in the R_c_ value. This suggested that the physical stability of these three samples was minimally impacted. As previously mentioned, a higher R_c_ value in a sample indicated that the sample exhibited greater resistance to transitioning from an amorphous solid to a crystalline form compared with samples with a lower R_c_ value. This indicated that the sample with a higher R_c_ value exhibited greater physical stability in comparison with the other samples with lower R_c_ values [8,17]. Moreover, amorphous solids typically exhibit high interfacial energy, making them susceptible to phase separation or agglomeration [35]. The feeding rate of the anti-solvent significantly impacted the stability of the interface. A feeding rate that is too rapid can destabilize the interface, thereby compromising the physical stability of the amorphous solids. Additionally, a rapid feeding rate can lead to a localized increase in temperature, resulting in a thermal effect. This thermal effect can negatively impact the physical stability of amorphous solids. Consequently, the feeding rate of the anti-solvent played a crucial role in determining the stability of amorphous solids through these two mechanisms. Consequently, the findings from the DSC analysis also suggested that a feeding rate of 5 mL/min can result in the creation of a more physically stable amorphous solid in comparison with the slower feeding rates of 0.1 and 0.5 mL/min and the faster feeding rates of 35 and 70 mL/min.

#### 2.1.3. Filtering Rate Analysis

**Figure 3:**
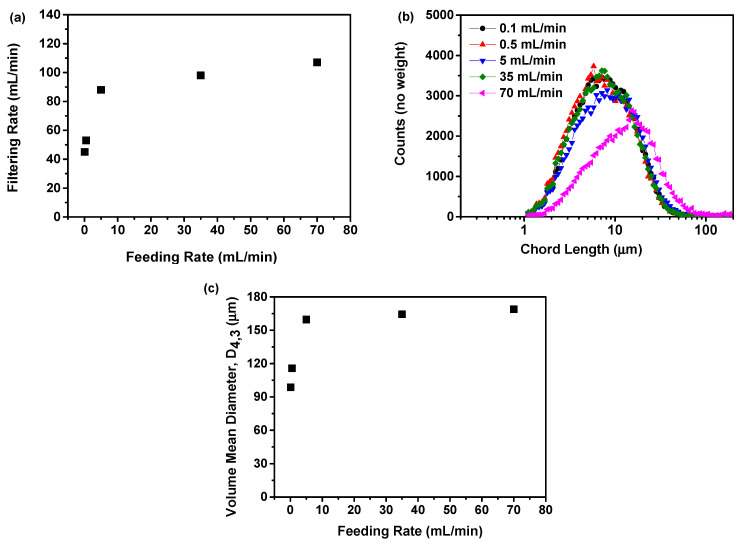
The influence of the feeding rate of the anti-solvent on the formation of nilotinib free base amorphous solids: (**a**) Filtering rate; (**b**) Particle size distribution; (**c**) Volume mean diameter, D_4,3_. (the agitation speed was fixed at 500 rpm, and the aging time was fixed at 10 min).

The influence of the feeding rate on the rate of precipitation suspension filtering was illustrated in Figure 3a. This study showed that the filtration rate rose in tandem with the increase in feeding rate. Specifically, an increase in the feeding rate from 0.1 to 0.5 mL/min resulted in a gradual rise in the filtration rate from 45 to 53 mL/min. Upon reaching a feeding rate of 5 mL/min, the filtration rate demonstrated a rapid increase to 88 mL/min. Moreover, as the feeding rate was raised to 35 and 70 mL/min, there was no significant increase in the filtration rate, with the filtration rates reaching 98 and 107 mL/min, respectively. This phenomenon may be attributed to an increased feeding rate, leading to the generation of larger solid particles within the precipitation suspension. The larger particles have the potential to increase the filtering rate, a finding that has been corroborated in prior studies [36].

To validate the aforementioned assumption, the particle size distribution was assessed using the FBRM, as shown in Figure 3b. The results indicated that with an increase in feeding rate, the chord length of the particles increased, while the particle count decreased. This means a higher feeding rate led to the production of larger particles while reducing the overall particle count. This can be verified by measuring the volume mean diameter based on the FBRM findings, as illustrated in Figure 3c. This study showed that the particle size increases with an increase in feeding rate. Specifically, an increase in feeding rate from 0.1 to 0.5 mL/min resulted in an increase in the volume mean diameter from 98.64 to 115.84 μm. However, upon reaching a feeding rate of 5 mL/min, the volume mean diameter exhibited a rapid increase to 159.65 μm. Nevertheless, as the feeding rate was further increased to 35 and 70 mL/min, there was no significant increase in the volume mean diameter, with the volume mean diameters measuring 164.38 and 168.87 μm, respectively.

Previous research has indicated that both the particle size and concentration of particles significantly impact the duration of the separation process [36,37]. So, in this study, a slow filtration rate could potentially extend the filtration process, thereby impacting the quality of the amorphous solids. During an extended filtration period, the amorphous solid has the potential to transform into a crystalline form. In addition, the feeding rate of the anti-solvent affected the microstructure of the amorphous solids [38]. A rapid feeding rate can introduce structural defects and accumulate stress within the amorphous solids, compromising their physical stability. Conversely, a slower feeding rate can promote the development of more consistent and stable amorphous solids. Accordingly, under the above mechanism, a feeding rate of 5 mL/min was determined to be more suitable in terms of filtration rate in the present study.

### 2.2. Effect of Agitation Speed

During the experimentation of agitation speed, the feeding rate was maintained at a constant rate of 5 mL/min, while the aging time was set at 10 min, allowing only the agitation speed to vary.

#### 2.2.1. PDF Analysis and PCA of PXRD Data

**Figure 4:**
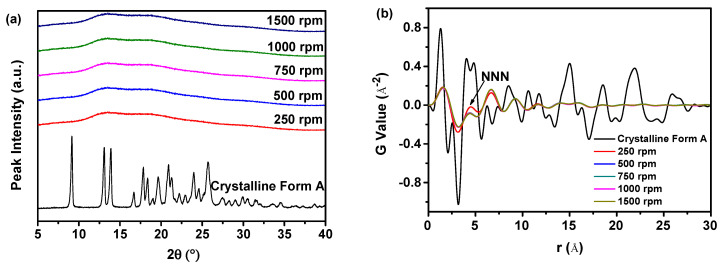

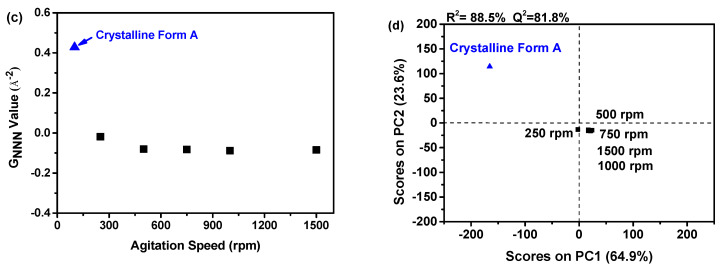
The influence of agitation speed on the formation of nilotinib free base amorphous solids: (**a**) PXRD patterns; (**b**) PDF trace (the NNN represents the next nearest neighbor atoms in the nilotinib free base solid samples); (**c**) G_NNN_ value; (**d**) PCA scores plot (the R^2^ represents the goodness of fit, while the Q^2^ represents the goodness of prediction). The crystalline Form A was used as a reference sample; the feeding rate was fixed at 5 mL/min, and the aging time was fixed at 10 min.

According to the data presented in Figure 4a, the PXRD patterns consistently showed the presence of a halo peak in all the samples of nilotinib free base, regardless of the agitation speed used, suggesting that they are in an amorphous form. Differentiating between these amorphous solid samples based on the PXRD patterns proved to be challenging. Nevertheless, the structural disparity in these amorphous solid samples could be discerned through the implementation of PDF analysis on PXRD data, as shown in Figure 4b. The amorphous solid sample prepared using an agitation speed of 250 rpm displayed the most pronounced fluctuation in the PDF trace and exhibited the highest peak height of NNN compared with the amorphous solid samples prepared at agitation speeds of 500, 750, 1000, and 1500 rpm. The peak height of NNN decreased as the agitation speed increased, suggesting that higher agitation speeds could effectively enhance the disorder of the amorphous solid samples.

Moreover, the degree of disorder was measured using the G_NNN_ value, as depicted in Figure 4c. The samples prepared with an agitation speed of 500 rpm exhibited a lower G_NNN_ value in comparison with those prepared with an agitation speed of 250 rpm, suggesting a higher degree of disorder in the samples prepared at an agitation speed of 500 rpm. It is important to note that the G_NNN_ values of the samples prepared with an agitation speed exceeding 500 rpm showed minimal variances, suggesting that an agitation speed of 500 rpm was adequate for attaining a heightened degree of disorder in the amorphous solid samples. Consequently, there is no need for agitation speeds higher than 500 rpm.

A more comprehensive evaluation of the differentiation between these amorphous solid samples was carried out by employing PCA with the data acquired from the PDF, as illustrated in Figure 4d. The study findings indicated a strong fit between the observed and predicted values, with the R^2^ measuring 88.5%. Furthermore, the Q^2^ was calculated to be 81.8%, indicating a high level of precision in forecasting future events. The results of the PCA analysis indicated that both the fitting and prediction were of high quality. Moreover, the PCA analysis results indicated that the samples could be categorized into three distinct groups. The first group comprised the reference sample (crystalline Form A), whereas the second group encompassed the sample prepared using an agitation speed of 250 rpm. The third group comprised four samples that were prepared using an agitation speed exceeding 500 rpm. The samples prepared with an agitation speed exceeding 500 rpm showed noticeable differences compared with those prepared with an agitation speed of 250 rpm, indicating structural distinctions. Moreover, the samples in the third group displayed a substantial deviation from the reference sample (crystalline Form A) along the PC1. This observation implied that the four samples exhibited a solid structure that is notably distinct from the reference sample (crystalline Form A). In addition, the samples prepared with an agitation speed exceeding 500 rpm displayed a comparable distance from the reference sample (crystalline Form A) on the PC1, suggesting a similar degree of disorder. This observation is consistent with previous studies [8,13,27].

Previous research revealed that the influence of agitation speed on the physical stability of amorphous solids is primarily manifested through its effect on the microstructure within the amorphous solids [39]. Changes in agitation speed can specifically alter the aggregation and dispersion of particles within the amorphous solids. At low agitation speeds, particles tend to agglomerate, forming larger aggregates. Conversely, at high agitation speeds, these particles are more evenly distributed. These alterations in microstructure directly impact the physical stability of amorphous solids. Therefore, the experimental findings suggested that elevating the agitation speed to 500 rpm resulted in the formation of a more physically stable amorphous solid in the present investigation.

#### 2.2.2. R_c_ Analysis of DSC Data

**Figure 5:**
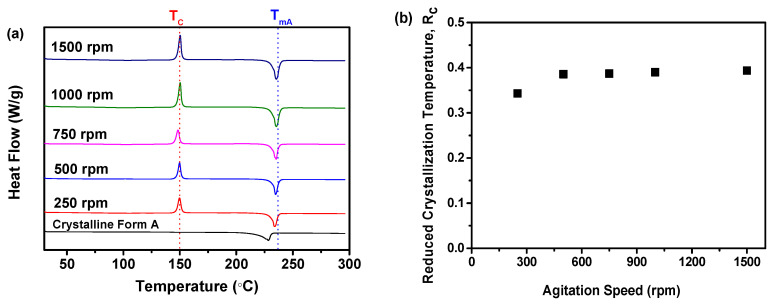
The influence of agitation speed on the formation of nilotinib free base amorphous solids: (**a**) DSC curves (the T_C_ represents the crystallization temperature of the amorphous solid samples, while the T_mA_ represents the melting temperature of nilotinib free base crystalline Form A); (**b**) Reduced crystallization temperature, R_c_ value. The crystalline Form A was used as a reference sample; the feeding rate was fixed at 5 mL/min, and the aging time was fixed at 10 min).

As illustrated in Figure 5a, the findings showed that all the samples, prepared at different agitation speeds, displayed a singular endothermic peak. This peak was associated with the melting temperature of nilotinib free base crystalline Form A (T_mA_, 235 °C) [25,26]. It was observed that all the samples exhibited an exothermic peak at 150 °C, attributed to the conversion of the amorphous solid state of nilotinib free base to crystalline Form A. This was supported by the T_c_ of the nilotinib free base crystalline Form A [8]. Furthermore, no significant variation in the T_c_ value was observed among the five samples collected at different agitation speeds. Furthermore, Figure 5b presents the fluctuation of the R_c_ value concerning agitation speed. The results indicated that the samples obtained at a lower agitation speed of 250 rpm demonstrated the lowest R_c_ value, whereas the samples prepared at an agitation speed exceeding 500 rpm exhibited higher R_c_ values. Moreover, the four samples prepared with an agitation speed exceeding 500 rpm showed a slight variation in the R_c_ value, suggesting a minor difference in their physical stability [8,17]. Mechanistically, agitation speed variations can significantly affect the interfacial energy of the amorphous solids [40]. Optimizing the agitation speed can effectively reduce the interfacial energy, thus improving the physical stability of the amorphous solids. Excessively high agitation speeds, however, can increase interface energy, ultimately compromising physical stability. Additionally, friction and collisions resulting from the agitation process can elevate local temperatures, inducing thermal effects. These thermal effects can significantly impact the physical stability of amorphous solids. Consequently, the DSC analysis can also verify that increasing the agitation speed to over 500 rpm can result in the production of more physically stable amorphous solids.

#### 2.2.3. Filtering Rate Analysis

**Figure 6:**
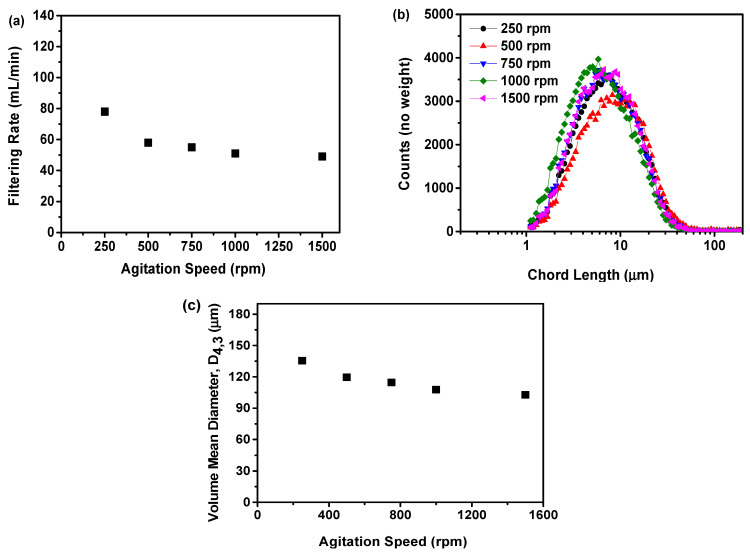
The influence of agitation speed on the formation of nilotinib free base amorphous solids: (**a**) Filtering rate; (**b**) Particle size distribution; (**c**) Volume mean diameter, D_4,3_. The feeding rate was fixed at 5 mL/min, and the aging time was fixed at 10 min.

The impact of agitation speed on the filtration rate is shown in Figure 6a. The study showed that an increase in agitation speed from 250 to 500 rpm resulted in a rapid decrease in the filtration rate from 78 to 58 mL/min. Nevertheless, as the agitation speed increased from 500 to 1500 rpm, there was no significant decrease in the filtration rate, which remained at 49 mL/min at 1500 rpm. The study results showed that the filtering rate decreased as the agitation speed increased. This phenomenon may be ascribed to a higher agitation speed, resulting in the production of smaller solid particles within the precipitation suspension. The existence of smaller particles can hinder the filtration process, resulting in a decrease in the filtration rate [36,37]. A previous study demonstrated that the formation of smaller struvite crystals was observed at increased agitation speeds [41].

To validate this assumption, the particle size distribution was assessed using the FBRM, as depicted in Figure 6b. An increase in agitation speed was found to be associated with a decrease in the chord length of the particles and an increase in particle counts. This indicated that an increased agitation speed led to the production of smaller particles, but a greater quantity of particles. This can be verified by measuring the volume mean diameter based on the FBRM findings, as illustrated in Figure 6c. This study showed that the average volume diameter decreases with an increase in agitation speed. The speed of agitation was directly correlated with the particle size in the suspension, and an increase in agitation speed resulted in a decrease in particle size, as indicated by previous research [36]. Moreover, this study demonstrated that agitation speed has a significant impact on the size and distribution of particles within amorphous solids. An appropriate agitation speed promoted the uniform distribution and refinement of particles, ultimately enhancing the physical stability of amorphous solids. Excessively high agitation speeds, however, can lead to the over-refinement of particles, potentially reducing physical stability. Accordingly, in terms of filtration rate, it was determined that an agitation speed of 500 rpm was more suitable in this study.

### 2.3. Effect of Aging Time

During the experimentation of aging time, the feeding rate was maintained at a constant rate of 5 mL/min, while the agitation speed was set at 500 rpm, allowing only the aging time to vary.

#### 2.3.1. PDF Analysis and PCA of PXRD Data

**Figure 7:**
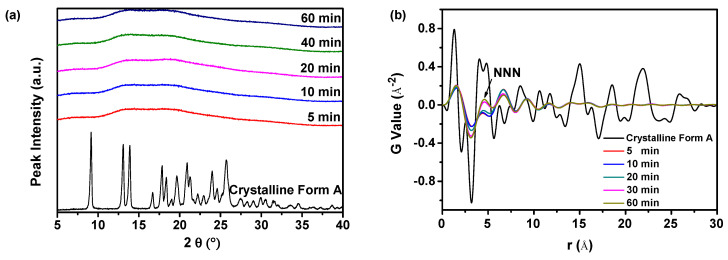

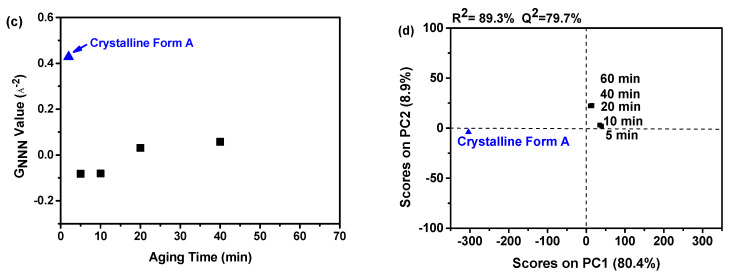
The influence of aging time on the formation of nilotinib free base amorphous solids: (**a**) PXRD patterns; (**b**) PDF trace (the NNN represents the next nearest neighbor atoms in the nilotinib free base solid samples); (**c**) G_NNN_ value; (**d**) PCA scores plot (the R^2^ represents the goodness of fit, while the Q^2^ represents the goodness of prediction). The crystalline Form A was used as a reference sample; the feeding rate was fixed at 5 mL/min, and the agitation speed was fixed at 500 rpm.

As illustrated in Figure 7a, the PXRD patterns indicated that the amorphous solid samples were successfully produced by altering the aging time from 5 to 60 min. Similarly, it was challenging to differentiate between these amorphous solid samples based on the PXRD patterns, as noted in reference [27]. Nevertheless, the application of PDF analysis to the PXRD data allows for the observation of variations in the structures of amorphous solids, as shown in Figure 7b. It was indicated that samples prepared with a longer aging time showed increased fluctuations in the PDF trace and a higher NNN peak height in comparison with samples prepared with a shorter aging time. Consequently, as the aging time decreased, the peak height of NNN decreased, suggesting that a more disordered amorphous solid could be produced with a shorter aging time. The prolonged aging time will increase Ostwald ripening, which in turn will cause the precipitation of more ordered amorphous solid samples or even crystalline forms [42].

Furthermore, the degree of disorder can be evaluated by employing the G_NNN_ value, as depicted in Figure 7c. Samples prepared with a shorter aging time demonstrated a lower G_NNN_ value, indicating that a higher degree of disorder can be attained by reducing the aging time. Previous studies have confirmed this conclusion, for example, it was revealed that the aging process in amorphous GeTe is characterized by the gradual transition of the local chemical arrangement towards a crystalline state [43]. Moreover, the observed aging effects suggest that the grinding of carbamazepine samples resulted in partial or complete disorder, as the morphology remained unchanged during storage [44]. Furthermore, the G_NNN_ value of the sample prepared with an aging time of 5 min showed similarity to that of the sample prepared with an aging time of 10 min. This indicated that extending the aging time from 5 to 10 min did not lead to a further increase in the degree of disorder in the amorphous solid samples.

PCA was performed on the PDF data to evaluate the difference between the amorphous solids, as illustrated in Figure 7d. The study findings indicated a high level of R^2^ at 89.3%, suggesting a robust correspondence between the observed and predicted values. Furthermore, the Q^2^ was calculated to be 79.7%, indicating a high level of precision in forecasting future results. The PCA results also suggested that both the fitting and prediction were of high quality. Furthermore, upon comparison with the reference sample (crystalline Form A), it was noted that all the samples could be classified into three distinct groups. The initial group was composed of the reference sample (crystalline Form A), whereas the subsequent group included samples that were prepared with an aging time of 5 and 10 min. The three groups consisted of samples that were prepared with aging times of 20, 30, and 60 min, respectively. The PCA score plot in Figure 7d illustrates that the sample with a shorter aging time exhibits a greater distance from the reference sample (crystalline Form A) along PC1 compared with the sample with a longer aging time. This observation implied that the sample exhibiting the greatest degree of disorder can precipitate when the aging time is set at 5 or 10 min.

Mechanistically, amorphous solids often exist in a high-energy state. The appropriate extension of aging time can facilitate enhanced rearrangement and interaction between the molecules of amorphous solids, ultimately leading to the improved stability of amorphous solids [45]. Excessively long aging times, however, can lead to the excessive rearrangement of the amorphous solids, which promotes the formation of a crystalline structure that can reduce its solubility. During the aging process, molecules in amorphous solids undergo rearrangement in search of a more stable conformation. If the interaction between the molecules of amorphous solids is strengthened during this rearrangement, it can promote crystallization, potentially compromising physical stability. Accordingly, the experimental results indicated that setting the aging time to 5 or 10 min led to the creation of a more physically stable amorphous solid in the current study.

#### 2.3.2. R_c_ Analysis of DSC Data

**Figure 8:**
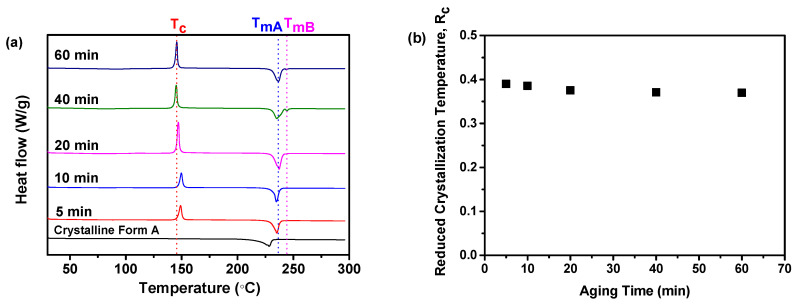
The influence of aging time on the formation of nilotinib free base amorphous solids: (**a**) DSC curves (the T_C_ represents the crystallization temperature of the amorphous solid samples, while the T_mA_ and T_mB_ represent the melting temperature of nilotinib free base crystalline Form A and Form B, respectively); (**b**) Reduced crystallization temperature, R_c_ value. The crystalline Form A was used as a reference sample; the feeding rate was fixed at 5 mL/min, and the agitation speed was fixed at 500 rpm.

As illustrated in Figure 8a, the findings showed that the samples produced with aging times of 5 and 10 min displayed a solitary endothermic peak. The observed peak corresponds to the melting temperature of crystalline Form A (T_mA_, 235 °C). Nevertheless, the samples prepared with an aging time of more than 20 min displayed two distinct endothermic peaks. The two observed peaks are attributed to the melting temperatures of crystalline Form A (T_mA_, 235 °C) and crystalline Form B (T_mB_, 245 °C) [25,26]. However, the peak at 245 °C was relatively small, indicating that a low quantity of crystalline Form B was formed during the heating process. Meanwhile, it was noted that all the samples displayed a solitary exothermic peak, which is indicative of the transition from the amorphous state to the crystalline Form A of nilotinib free base, as evidenced by the T_c_ of the nilotinib free base crystalline Form A [8]. The T_c_ values of the samples prepared with aging times of 5 and 10 min exceeded those of samples aged more than 20 min. Furthermore, there was no noticeable difference in the T_c_ value between the two samples aged for 5 and 10 min.

Furthermore, Figure 8b demonstrates the fluctuation of the R_c_ value concerning aging time. It was revealed that the sample prepared with a shorter aging time displayed the highest R_c_ value, whereas the samples prepared with a longer aging time showed lower R_c_ values. However, the samples prepared with aging times of 5 and 10 min show only a slight difference in the R_c_ value, suggesting a minor variation in their physical stability. Mechanistically, amorphous solids undergo a glass transition during aging, transitioning from a viscous state to a solid state. This transition involves the reduction of amorphous solids’ molecule mobility and the enhancement of intermolecular interactions, potentially leading to improved physical stability [46]. Additionally, aging time facilitates the structural relaxation of amorphous solids, which refers to the gradual movement and rearrangement of amorphous solid molecules while maintaining a solid state. This relaxation reduces internal stress within the amorphous solids, thereby enhancing their physical stability. As aging time increases, amorphous solids exhibited changes in their physical properties, directly impacting their overall physical stability. Thus, the DSC analysis can also offer additional confirmation that a physically more stable amorphous solid can be attained when the aging time does not exceed 10 min.

#### 2.3.3. Filtering Rate Analysis

**Figure 9:**
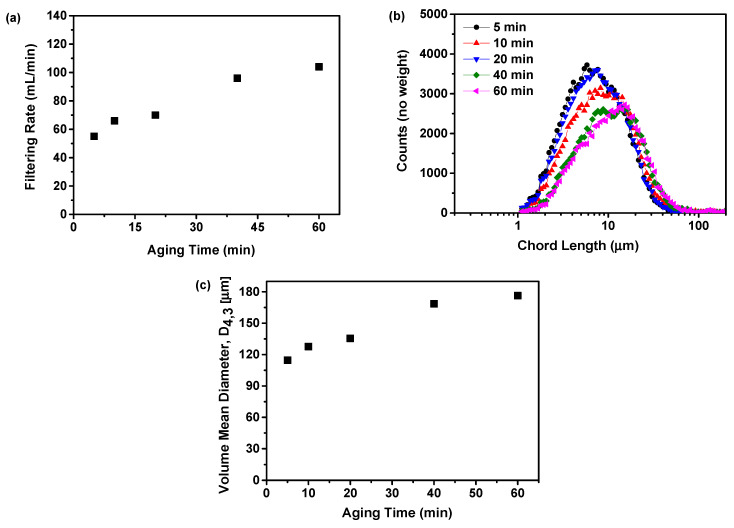
The influence of aging time on the formation of nilotinib free base amorphous solids: (**a**) Filtering rate; (**b**) Particle size distribution; (**c**) Volume mean diameter, D_4,3_. The feeding rate was fixed at 5 mL/min, and the agitation speed was fixed at 500 rpm.

The impact of aging time on the filtration rate is present in Figure 9a. This study showed that an increase in aging time from 5 to 10 min resulted in a rise in filtration rate from 55 to 66 mL/min. However, at aging times of 20, 40, and 60 min, the filtration rate exhibited a rapid increase to 70, 96, and 104 mL/min, respectively. The study findings indicated that the filtration rate rose in correlation with the aging time. This phenomenon could be attributed to an extended aging time, leading to the generation of larger solid particles within the precipitation suspension. These larger particles have the potential to increase the rate of filtration [36].

To validate this result, the particle size distribution was assessed using the FBRM, as depicted in Figure 9b. It was observed that with an increase in aging time, the chord length of the particles increased, whereas the particle count decreased. This indicated that an extended aging time led to the formation of larger particles while reducing the overall particle count. The phenomenon can be attributed to the disintegration of small particles and the enlargement of larger particles. This can be verified by measuring the volume mean diameter based on the FBRM findings, as illustrated in Figure 9c. The results indicated that the volume mean diameter increases with a longer aging time. The duration of aging is directly correlated with the particle size in the suspension. An increase in aging time results in an increase in particle size, as indicated by previous research [42].

The results indicated that if the aging time was too short, the particle size distribution became uneven. Conversely, excessive aging times can lead to particle growth and agglomeration, making it challenging to achieve ultrafine particles. Therefore, selecting the appropriate aging time is crucial for managing particle size and distribution. The precise optimal aging time varied based on factors such as material type, process conditions, and desired particle characteristics. Consequently, it was essential to determine the optimal aging time through rigorous experimentation and process optimization. Therefore, based on considerations of physical stability and filtration rate, an aging time of 10 min was deemed more appropriate for this study.

## 3. Materials and Methods

### 3.1. Materials

The nilotinib free base crystalline Form A solid was supplied by Heryi Pharma (purity of ≥98%, Anhui Heryi Pharmaceutical Co., Ltd., Tianchang, China). DMSO was procured from Titan (purity ≥ 99%, Shanghai Titan Technology Co., Ltd., Shanghai, China). Deionized water was acquired using a Millipore ultrapure water system (Applied Membranes Inc., Vista, CA, USA).

### 3.2. Preparation of Nilotinib Free Base Amorphous Solids

**Figure 10:**
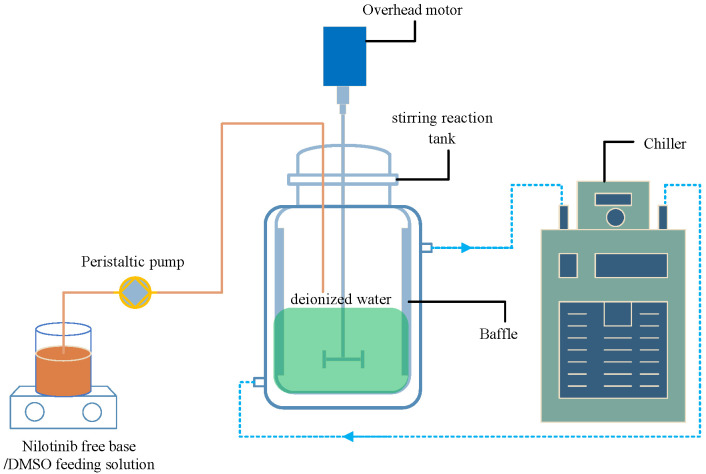
Experimental setup for the preparation of nilotinib free base amorphous solids.

As shown in Figure 10, an appropriate amount of solid nilotinib free base crystalline Form A was fully dissolved in DMSO (used as a solvent) at a temperature of 70 °C, leading to the creation of a 10 mL (0.27 mol/L) solution of nilotinib free base. The solution was subsequently filtered to remove any solid impurities. Subsequently, a suitable quantity of deionized water, serving as an anti-solvent, was introduced into a stirring reaction tank equipped with four baffles (S300, Beijing Century Senlang Experimental Instrument Co., Ltd., Beijing, China). The deionized water was cooled to 5 °C with the use of a chiller (KGDH-2030, Nanjing Kenfan Electronic Technology Co., Ltd., Nanjing, China). The nilotinib free base solution was fed into the deionized water in the stirring reaction tank using a peristaltic pump (BT100FC, Baoding Rongbai Constant Flow Pump Manufacturing Co., Ltd., Baoding, China) at varying feeding rates of 0.1, 0.5, 5, 35, and 70 mL/min. To promote the process of anti-solvent precipitation, the agitation speeds of the overhead motor used were 250, 500, 750, 1000, and 1500 rpm, respectively. The ratio of anti-solvent to solvent volume was set at 40. The low solubility of nilotinib free base in deionized water led to the rapid precipitation of a solid suspension containing particles of nilotinib free base when mixed with deionized water. The suspension underwent continuous stirring and aging for 5, 10, 20, 40, and 60 min, respectively, following the completion of the feeding process. The wet filter cake of nilotinib free base was obtained through a filtration process using a circulating water vacuum pump (SHZ-III A, Gongyi Ruide Instrument Equipment Co., Ltd., Gongyi, China) and qualitative filter paper. During the filtration process, the wet filter cake was rinsed with 50 mL of deionized water to eliminate the DMSO solvent from the wet filter cake of the nilotinib free base. Subsequently, the rinsed wet filter cake underwent drying in a vacuum oven (DHG-9055A, Wujiang Yonglian Machinery Equipment Factory, Wujiang, China) at 40 °C for 18 h. Ultimately, dried samples of nilotinib free base with a water content of about 3.5% were acquired. The dried samples were then transferred into glass bottles with caps and stored at room temperature before measurement. Each experiment was repeated three times.

### 3.3. Characterization of Nilotinib Free Base Solid Samples

#### 3.3.1. Powder X-ray Diffraction (PXRD)

The PXRD instrument (Bruker D8 with semiconductor detectors, Bruker, Karlsruhe, Germany) was employed to conduct measurements on the dried samples at ambient temperatures. The PXRD measurement parameters employed in this study included the use of Cu Kα radiation with a wavelength of 1.54 Å. The tube voltage was set at 100 kV and the tube current was set at 80 mA. A consistent scanning increment of 0.02° and a scanning velocity of 6°/min were upheld. The diffraction angle range for the scans was configured to span from 5 to 40°. Each sample was subjected to three measurements.

#### 3.3.2. Differential Scanning Calorimetry (DSC)

The dried samples were subjected to thermal analysis using a DSC instrument (Q2000 TA Instruments, New Castle, DE, USA), and each sample was subjected to three measurements. The temperature range extended from 30 to 300 °C, with a consistent heating rate of 10 °C/min. Approximately 5 mg of the dried samples were enclosed in a sealed disk (T161116, TA Instruments, New Castle, DE, USA) and subjected to nitrogen purging at a flow rate of 50 mL/min while being heated. The glass transition temperature (T_g_), crystallization temperature (T_c_), and melting temperature (T_m_) were determined based on the data obtained from DSC measurement. Subsequently, the reduced crystallization temperature (R_c_) was determined using the formula established in a previous study [18]. The R_c_ value was defined as follows:

$$R_{c}=\frac{T_{c}-T_{g}}{T_{m}-T_{g}}$$

#### 3.3.3. Focused Beam Reflectance Measurement (FBRM)

The FBRM instrument (FBRM G400, Mettler Toledo, Columbus, OH, USA) was employed to continuously monitor the particle size of the nilotinib free base precipitates. The FBRM probe was directly integrated into the stirring reaction tank. The particle size in the stirring reaction tank was monitored at 10 s intervals during the feeding of the feed solution into the stirring reaction tank. Each experiment was repeated three times.

### 3.4. Filter Test

The sample suspension was filtered using a vacuum pump (DOA-P704-AC, GAST, Chicago, IL, USA) at a constant volume of 410 mL. The duration from the initiation of filtration, until no filtrate drops passed through the filter paper, was recorded as the filtration time. The filtration rate was determined by dividing the volume of the suspension by the duration of the filtration process. Each experiment was repeated three times.

### 3.5. Pair Distribution Function (PDF)

The PDF analysis was conducted by applying Fourier transformation to the PXRD data using the freeware program PDFgetX3 (Version 2.2.1, Columbia University, New York, NY, USA). Detailed parameter settings for PDF analysis with this software can be found in the study conducted by Juhás et al. [47]. The program is accessible via the URL: https://www.diffpy.org/products/pdfgetx.html (accessed on 4 January 2024). Each analysis was repeated three times.

### 3.6. Principal Components Analysis (PCA)

This study utilized PCA as a statistical technique to aid in comprehending the observed variations in the PXRD patterns and PDF data of distinct samples. As delineated in the investigation carried out by Karmwar et al., the preprocessing and scaling of PCA were executed utilizing SIMCA 15 software (Version 15.0, Sartorius, Göttingen, Germany) [48]. During the PCA analysis, a specific range of interatomic distances in the PDF, namely 0–15 Å, was selected, and the PCA data for each solid sample was subsequently obtained. Each analysis was repeated three times.

## 4. Conclusions

Amorphous solids are susceptible to physical instability and may experience crystallization, resulting in decreased solubility and dissolution rates of the compound. This study aimed to generate physically stable amorphous forms of the anticancer medication nilotinib free base by manipulating various parameters associated with amorphous precipitation. The parameters encompassed the feeding rate, agitation speed, and aging time. The findings indicated that the amorphous solid samples demonstrated enhanced physical stability and filtration rates under certain conditions. The greatest physical stability and most favorable filtration rate were noted at a feeding rate of 5 mL/min, an agitation speed of 500 rpm, and an aging time of 10 min. Moreover, the comprehensive methodology involving the performance of PDF analysis and PCA on PXRD data, along with the computation of the R_c_ value from DSC data, enabled the assessment of the physical stability of amorphous solids. Furthermore, the physical stability of amorphous solids was primarily influenced by particle size and distribution, molecular interactions, microstructure, surface area, and interfacial energy. Notably, the parameters involved in the anti-solvent precipitation process, such as the feeding rate of the anti-solvent, agitation speed, and aging time, have a significant impact on these factors. Consequently, they directly affected the physical stability of amorphous solids. Hence, this study comprehensively elucidated the mechanistic influence of these operational parameters on the physical stability of amorphous solids during the anti-solvent precipitation process. The effect of the feeding rate of the anti-solvent on the physical stability of amorphous solids was primarily attributed to nucleation and growth, the mixing of anti-solvent and solvent, microstructure, interfacial stability, and thermal effects. The impact of agitation speed on the physical stability of amorphous solids was primarily attributed to factors such as particle size and distribution, microstructure, interfacial energy, and thermal effects. Finally, the effect of aging time on the physical stability of amorphous solids was primarily attributed to molecular rearrangement, crystallization, glass transition, and structural relaxation. Therefore, during the anti-solvent precipitation process of amorphous solids, various factors cumulatively affected the physical stability of the amorphous solids, ultimately leading to the attainment of physical stability under different preparation conditions. Therefore, in the actual production process, it was particularly important to optimize the preparation conditions of amorphous solids.

## Data Availability

Data are contained within the article.
